# Investigation on the Micro Cutting Mechanism and Surface Topography Generation in Ultraprecision Diamond Turning

**DOI:** 10.3390/mi13030381

**Published:** 2022-02-27

**Authors:** Quanhui Wu, Guoda Chen, Qinglin Liu, Baisong Pan, Wanqun Chen

**Affiliations:** 1 Key Laboratory of Special Equipment Manufacturing and Advanced Processing Technology, Ministry of Education, Zhejiang University of Technology, Hangzhou 310023, China; gchen@zjut.edu.cn (G.C.); panbsz@zjut.edu.cn (B.P.); 2 School of Mechatronics Engineering, Harbin Institute of Technology, Harbin 150001, China; chwq@hit.edu.cn; 3 Zhejiang Zomax Transmission Co., Ltd., Wenling 317513, China; irm@zomaxtransmission.com

**Keywords:** ultraprecision diamond turning, aerostatic bearing spindle, coupling vibrations, micro cutting mechanism, surface topography

## Abstract

Revealing forming mechanism of workpiece surface topography plays an important role in improving ultraprecision turning. In this paper, the forming mechanism of the turning workpiece surface topography is analyzed and verified by the theoretical simulation and experiment respectively. First, the factors directly related to the turning process are analyzed, and a volumetric error model is built and discussed, which considered geometric errors, tool geometry, spindle vibrations, feed rate, cut depth, and feed system position change. The vibration mechanism and laws of the spindle system under multi-field coupling is analyzed, and the effect of the spindle axial vibration on the turning surface topography is studied. In addition, influence of coupled vibrations on the turning surface texture is analyzed, and an equivalent machining model is constructed to identify crucial geometric errors of the workpiece surface topography. Finally, a homemade ultraprecision machine tool system is built and used for turning the workpiece surface, and the tested results of the surface topography demonstrate *Ra* is better, 10 nm and *Rv* is better, 20 nm. The end face of the workpiece forms periodically fluctuating wave and ripple patterns, and the comparison between theoretical analysis and experimental detection of the surface topography is verified.

## 1. Introduction

In the field of optical applications, as the core component of the optical system, optical mirror surface quality on optical components has become important in manufacturing critical components. With increasing demands on high-performance and functional products, optical mirrored parts have become bottleneck issues for specific components to meet the ultraprecision machining requirements. As further machinery technology development, single-point diamond turning (SPDT) has become a key step in the ultraprecision turning process chain, rapidly. In SPDT, different factors may significantly affect surface texture generation, and limit the machining process outcome [1]. Subsequently, in order to obtain high quality optical surfaces, influencing factors on surface profile generation should be introduced and comprehensively discussed, which is used to optimize turning parameters and reveal the forming mechanism of the workpiece surface.

An aerostatic bearing spindle (ABS) is an important part in ultra-precision machine tools, and its dynamic performance plays a major role in the workpiece surface quality directly, which mainly causes mid-spatial waviness errors on workpiece surface topography. Ning et al. [2] designed a large amplitude umbrella surface using a slow tool servo (STS) of SPDT, and obtained the micro-nano structure of the workpiece surface. As micro-nano surface shows many excellent characteristics in optics, biology, and so on, the ultraprecision technology has been applied to various fields [3,4,5,6,7,8]. Li et al. [9] proposed the machining theory of free-form surfaces and verified the validity theory in STS. Wang et al. [10] proposed a comprehensive tool-path generation strategy including the trajectory interpolation, and provided progressive additive lens surface experiments.

Ultra-precision turning is one of the most basic machining methods in the mechanical manufacturing, which is widely used to turning the ultraprecision surface quality components in the fields of aerospace and automobile. Turning vibration generated during machining parts is a process that seriously affects chip formation, and ultimately reduces the workpiece surface quality [11,12]. During ABS rotation, the spindle shaft is supported through the pressurized air in the air gap, and a direct drive structure between the motor and the spindle shaft is adopted. Due to the inherent reasons such as cogging effects caused by the motor assembly errors, the electromagnetic field characteristics will inevitably affect the spindle vibration [13]. In order to study the influence of the motor characteristics on the rotation accuracy of ABS, Usman [14] used the axial displacement target ball method to detect the spindle vibration errors. Zhang et al. [15] adopted the hydrostatic spindle system driven to conduct turning experiments, and found ripples in the workpiece surface which is related to the number of motor poles. From the above research, the motor characteristics have a direct impact on the performance of the spindle system.

As the main functional part in the turning process, the static and dynamic performance of ABS has great influence on turning workpiece surface quality. In order to fully analyze the characteristics, Cui et al. [16] used dynamic grid technology and finite element method to quantitatively study the impact of manufacturing errors on the operating accuracy of ABS. Huang et al. [17] studied the variation of the spindle average sliding axis error, and found the spindle average sliding axis error increasing with the spindle speed [18]. Chen et al. [19] analyzed the relationship between the vibration frequency during the machining process and the spatial vibration frequency of the machined surface. In order to improve the accuracy of thermal-structural modeling, Liu et al. [20] proposed a geometric mechanical thermal model of the spindle system based on a closed-loop iterative modeling method. Su et al. [21] constructed the fluid-solid coupling model of ABS, and studied the influence of temperature rise on the stiffness and bearing capacity of the air film. Previous research comprehensively considered the mutual influence of electromagnetic effects, static pressure characteristics, servo control, and mechanical characteristics according to the design principles [22].

In common, the surface quality of ultraprecision machining is detected by an optical detection instrument, and the impact on the surface quality during the cutting process is analyzed through the detection results. In order to use ultra-precision turning technology to obtain optical mirror elements, it is very important to study the surface quality of ultra-precision turning mirror [23,24]. Pawlus [25] discussed the analysis and usefulness of the material ratio curve, considering the practical significance of the calculation parameters. Pp [26] analyzed the statistical dependencies among 17 non-dimensional parameters on the basis of the linear coefficient of correlation. Niu [27] conducted metrology assessments on the workpiece surface roughness, topography, texture, and defects/features using a ZYGO surface profiler. Swojak [28] presented a method for determining the uncertainty of measurement, and applied the procedure of calibration and estimation of static characteristics coefficients. Krolczyk [29] focused on the parametric and nonparametric description of surface topography after turning in the dry and MQCL conditions. 

Ultra-precision turning technology is widely used in the manufacture of large metal mirrors. However, the poor surface quality of ultra-precision turning causes serious diffraction effects. It is very important to analyze the surface quality and surface-forming mechanism. The forming mechanism of ultra-precision turning surface topography is a complicated forming process, which is affected by the tool parameters, the spindle performance, tool geometric errors, vibrations between tool and workpiece surface, etc. In this paper, the surface topography forming mechanism of ultra-precision turning process is studied to analyze the multi-field coupling characteristics of the spindle system, and comprehensively study the influence of electromagnetic, unbalanced shaft rotation, and dynamic performance on aerostatic bearing spindle performance. Then, the mathematical model of the workpiece surface topography is established and simulated. Finally, the experimental inspection of the topography of the turning end face is carried out to verify the correctness of the theoretical analysis using a homemade machine tool under actual working conditions.

## 2. Theoretical Analysis of the Workpiece Surface Topography

**Figure 1:**
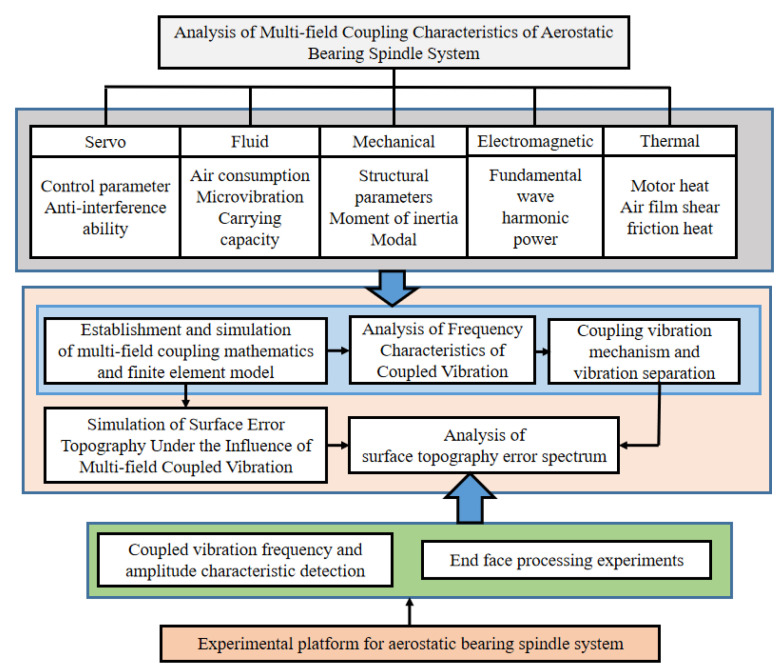
Flow charts of simulation and experiment of the workpiece surface topography.

In order to analyze the multi-field coupling characteristics of ABS in detail, research flow chart of the workpiece surface topography is shown in Figure 1. Based on the multi-field coupling characteristics of ABS, the multi-field coupling mathematical model and physical model of ABS are established, and the coupling model is simulated and analyzed. ABS model is used to study the influence of unstable factors such as mechanical, static pressure, servo, electrical, and thermal characteristics on the dynamic performance, including the different number of motor pole slots, motor rotor eccentricity, motor rotor imbalance, air supply pressure fluctuations, and other unstable factors. Through a combination of the theoretical analysis and experiment method, the vibration mechanism during the machining process is studied, and multiple fields are revealed in the generation mechanism of the vibration frequency domain under the coupling action, which separates the multi-field coupling vibrations, realizes the optimization under the multi-field coupling action of mechanical, electromagnetic, servo control, and static pressure, etc. 

During machining process of the workpiece surface, due to the movement errors between the tool tip and the workpiece surface, micro ripples will appear on the machined workpiece surface, which will seriously affect the workpiece surface accuracy. In order to deeply understand the formation process of on the workpiece surface topography during turning, it is necessary to establish an accurate theoretical model to describe the geometric relationship between the tool tip movement and the workpiece surface. To predict the workpiece surface topography and optimize the turning process, there is a lack of theoretical models that comprehensively consider different scales. The relative motion errors of the turning process is considered, and a theoretical model of the turning surface topography is established, revealing the formation mechanism of surface micro-waviness and the cumulative process of phase shift. Due to the small rotational speed error of the turning tool, the phase shift inevitably appeared in the entire machining process, resulting in umbrella-like micro-waves appearing on all machining surfaces. Moreover, small changes in phase shift can also cause significant differences in surface roughness, which indicates that phase shift is also an important variable for optimizing ultra-precision turning process. Finally, considering the geometric shape and relative motion error, a theoretical formation model of the flat surface topography is established.

Because of the machining accuracy requirement, the small errors of the turning tool movement have significant effects on the surface quality of micrometer and nanometer level, which not only seriously affects the performance and the product life cycle, but also increases the workload of the subsequent polishing. This seriously affects the production efficiency of the product. The corresponding geometric model of micro-corrugation, considering the relative motion trajectory is established, turning profile and phase shift of the tool tip to reveal the mechanism and evolution of micro-corrugation, so as a theoretical basis for subsequent theoretical modeling and optimization of turning process is provided. In the end face machining process, generally a very small cutting depth is used to achieve the micro-removal material. Machining parameters can determine the relative cutting position and tool path of the turning tool relative to the workpiece surface, which can affect the workpiece surface topography. Therefore, the machining parameters, the cutting profile geometry of the turning tool, and the relative position errors between the tool and the workpiece surface have a great influence on the surface formation.

In order to make further clarification and discussion, the analysis of the surface profile should be carried out. The specific analysis is as follows: the surface roughness causes are predicted by cutting tool geometry, feed rate, cutting depth etc. The primary profile causes directly depend on numerous factors such as the condition of the machine, cutting tool, parameters etc. The errors of the form deviation in the workpiece surface quality are caused by errors in the guideways of the machine tool, workpiece deflection, material deflection, hardening distortion, wear. The errors of the waviness in the workpiece surface quality are caused by errors in the off-center clamping, form or concentric deviation of a turning cutter, machine or tool vibrations. In addition, the errors of the roughness in the workpiece surface quality are caused by the cutting tool edge shape, feed or depth of cut of the tool. 

**Figure 2:**
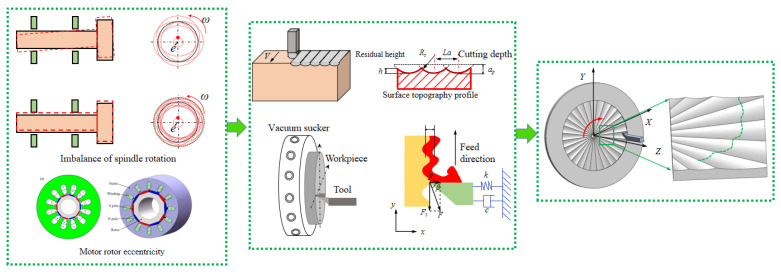
Schematic diagram of the ultra-precision turning.

During ultraprecision turning process, the various factors from the machine, tool, process variables, and the material properties affect the surface generation including the surface topography, texture, and roughness, which will have a great impact on the surface quality and accuracy of the workpiece surface. Therefore, it is necessary to establish a corresponding theoretical model to study the influence of the relative movement error of the tool on the workpiece surface formation, so as to optimize the turning process and improve the accuracy of the turning surface process. In the actual turning process, the movement errors between the tool tip and the workpiece surface cause periodic changes in the position of the machining tool, resulting in excess relative movement which has an important impact on the surface quality, produces periodic changes on the workpiece surface, and the micro ripples are shown in Figure 2. During the machining process, the micro ripples formed on the workpiece surface are affected by the rotation speed of the machining tool tip, the feed speed, and the rotation speed of the workpiece, resulting in the formation of surface micro ripples with different geometric patterns. In order to study the law of surface micro-waviness changing with these process parameters and the different texture features presented, it is necessary to analyze the movement trajectory of the turning tool relative to the workpiece surface, the distribution of the cutting points of the turning tool on the workpiece surface and the law of corrugation movement.

**Figure 3:**
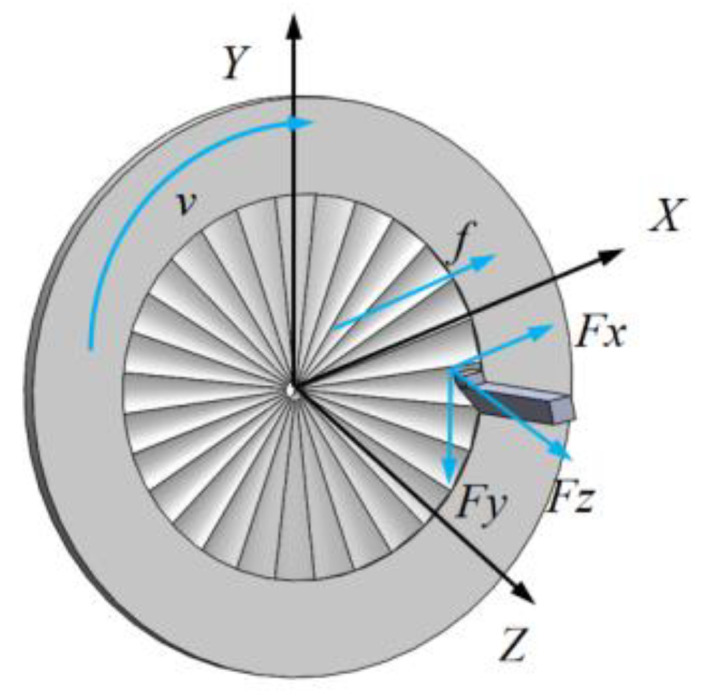
Surface waviness formation process.

The tool tip is used to remove the material on the workpiece end face, and the tool tip will mirror the contour between the tool tip and the workpiece surface along the tool path. Under the constant-speed cutting condition where the spindle speed and feed rate are constant, the tool path of the turning face is the result of the combined action of the workpiece constant rotation and the tool constant feed, which is expressed as Equation (1) in polar coordinates. In machining process, turning force is an important factor affecting the cutting process. Turning force directly affects the results of cutting heat, workpiece surface quality, tool wear, and so on. Therefore, the turning force is of a great significance for analyzing the workpiece surface topography of the spindle speed disturbance. The turning force acts on the rake face near the cutting edge, and its size and direction change during the turning process. In order to facilitate the analysis and study of the turning force, the turning force *F* is often based on the feed direction, depth between machining direction and main motion speed. The direction is decomposed into three components *Fx*, *Fy*, and *Fz* perpendicular to each other on the space rectangular coordinate axis *x*, *y,* and *z*, which is shown in Figure 3. After decomposition, the resultant cutting force is obtained and is expressed as Equation (2).

(1) $$\{\begin{array}{c} \rho =vt\\ \theta =\frac{2\pi n}{60}t \end{array}$$

(2) $$F=\sqrt{F_{x}^{2}+F_{y}^{2}+F_{z}^{2}}.$$

where *ρ* represents the distance between the tool tip and the center of the circle; *θ* represents the angle difference between the tool tip and the starting angle; *n* represents the spindle speed; *t* represents the cutting time; and *F* represents the machining force.

Due to the turning self-excited and external interference, the machining speed and the feed rate are periodically changed, and the machining force also changes periodically. Therefore, the relative displacement in the axial direction also changes periodically, and the machining force changes. The rigidity in the axial direction of the disc-shaped part is the weakest in the machining process, and the tool tip and the workpiece can be regarded as a spring damping system in the axial direction of the part. When the turning force of the spindle system changes periodically, the relative displacement outputs between the tool tip and the workpiece change periodically. Taking into account the relative displacement changes, the constant-speed cutting tool trajectory is modified, and periodic fluctuations in the axial direction are added on the basis of the plane spiral, which represents the relative displacement change between the tool tip and the workpiece surface in this direction, so the trajectory equation can be expressed as Equation (3).

(3) $$\{\begin{array}{c} n(t)=n_{0}+n_{A}\sin(2\pi ft)\\ v_{f}=n(t)\cdot f_{v}\\ \rho =v_{f}t\\ \begin{array}{c} \theta =\frac{2\pi n(t)}{60}t\\ z=A_{z}\sin(2\pi ft+\phi ) \end{array} \end{array}$$

where *z* represents the vibration of the tool path in the axial direction, *n_0_* represents the basic speed of the spindle, *n_A_* represents the amplitude of the spindle speed disturbance, and *f* represents the frequency of spindle speed disturbance.

**Figure 4:**
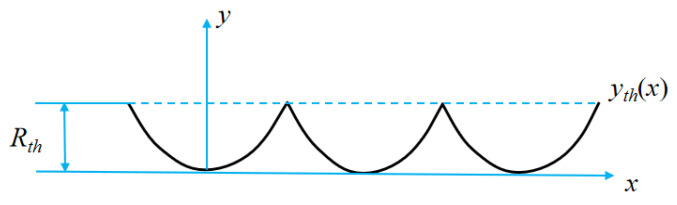
Surface profile of workpiece in theoretical conditions.

Compared with the constant-speed cutting tool trajectory, the spindle speed perturbs the cutting tool trajectory periodically fluctuates in the axial direction, so the workpiece surface topography will be affected by cyclical influence. Under ideal turning conditions, the machining process is a process in which the tool contour is copied to the machining workpiece surface along the tool trajectory, and the final process is to form the surface topography. Tool trajectory refers to the motion trajectory between the tool tip relative to the workpiece surface. Under the constant-speed cutting condition where the spindle speed is constant, the tool trajectory of end face turning is the result of the combined action of the workpiece’s uniform rotation and the tool’s uniform feed. The tool tip trajectory is on a plane. From a geometric viewpoint, the cutting surface is a Boolean subtraction of the tool geometry along the tool path on the machined surface. Assuming that the cross-sectional profile of the tool tip is circular ideally, the radial cross-sectional profile of the workpiece is the outer edge line of the circular profile combination, as shown in Figure 4. The cross-sectional profile can be expressed as Equation (4), and the cross-sectional height profile can be expressed as Equation (5).

(4) $$y_{th}(x)=\{\begin{array}{c} r_{e}-\sqrt{r_{e}^{2}-x^{2}},\;0\leq x\leq f/2.\\ r_{e}-\sqrt{r_{e}^{2}-{\left(f-x\right)}^{2}},\;f/2\leq x\leq f. \end{array}$$

(5) $$R_{th}=\frac{f^{2}}{8r_{e}}$$

where *y_th_* represents the radial cross-sectional profile of the workpiece under ideal conditions, *r_e_* represents the radius of the tool tip, *f* represents the feed per revolution of the tool, *x* represents the radial coordinate of the workpiece, and *R_th_* represents the theoretical surface height.

**Figure 5:**
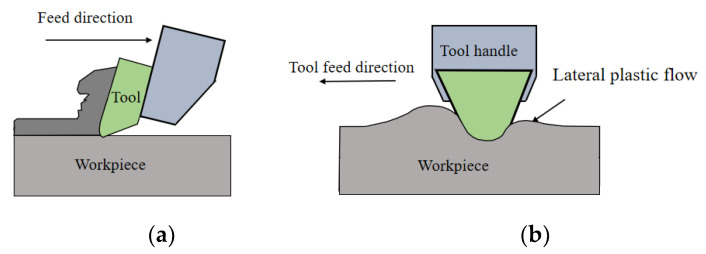
Schematic diagram of lateral plastic flow in metal machining process [30]. (**a**) Cutting direction, (**b**) tool feed direction.

The above model expression is based on the machining process under ideal conditions, but the actual results show that the profile height of the actual process section is often larger than the predicted model. The metal machining process is accompanied by the process such as elastic deformation, plastic deformation, cracking and separation of the metal material. The plastic deformation of the metal workpiece will affect the formation of the surface topography. Lateral plastic flow is a phenomenon in which the cutting edge and the metal material squeeze during the machining process, and the metal material is subjected to high pressure and plastic flow on both sides of the cutting edge. As shown in Figure 5, a part of the material rubs, deforms, and flows out along the rake surface, and a part of the material goes down around the corner between the cutting edge and flows out along the flank to form a machined surface. The material in the middle makes a lateral plastic flow relative to the tool forming a lateral plastic flow. The lateral plastic flow affects the undulation of the workpiece surface profile, and therefore also affects the workpiece surface roughness.

The lateral plastic flow surface model is based on the analysis principle of the metal scratch experiment. The scratch experiment is mainly used to study the mechanical properties of the metal surface. The surface form and scratch hardness mainly depend on the rheological properties of the metal material, the friction of the contact surface, and the tool geometry. In the scratch experiment, combined with the plastic pressure and elastic pressure characteristics of the metal, the rheological property *x* is the ratio of the plastic pressure to the elastic pressure, and the rheological property of the metal can be expressed as Equation (6).

(6) $$\{\begin{array}{c} \varepsilon_{p}=\cot\theta \\ \varepsilon_{e}=\frac{\sigma_{y}}{E}\\ x=\frac{\varepsilon_{p}}{\varepsilon_{e}}=\frac{E\cot\theta }{\sigma_{y}} \end{array}$$

where *ε_p_* represents the plastic pressure of the metal material, *ε_e_* represents the elastic pressure of the metal, *θ* represents the half-vertex angle of the scratch test tool, *E* represents the Young’s modulus of the test metal, and *σ_y_* represents the yield strength of the test metal.

Considering the above two factors comprehensively, the periodic vibrations between the tool path in the direction perpendicular to the workpiece surface make the bottom of the surface profile periodically change. At the same time, the lateral plastic flow changes periodically due to the change of cutting force, resulting in the peak profile. Based on the influence of the above-mentioned lateral plastic flow and relative vibration, a topography model based on Fourier expansion is proposed. The expression of the topography model under normal cutting conditions is expressed as Equation (7).

(7) $$\{\begin{array}{c} y(x)=(1+k)y_{th}(x)\\ k=\frac{R_{p}}{R_{th}} \end{array}$$

The above formula indicates that the actual turning profile is obtained by multiplying (*1* + *k*) on the basis of the theoretical profile, where *k* represents the ratio of the surface height between the lateral plastic flow profile and the theoretical profile surface height. The theoretical contour surface height *R_th_* is related to the tool tip radius and the feed per rotation. The theoretical contour surface height *R_th_* remains unchanged under the condition that the tool tip radius and the feed per revolution remain unchanged, and *R_p_* is the lateral plastic flow contour surface height. The variables in the formula include the Young’s modulus *E* of the metal, the half apex angle of the tool, the undeformed chip thickness *t*, the chip thickness *w*, the normal shear angle of the tool *n*, the vertical the cutting force *Fy* on the workpiece surface, and the turning force *F_z_* in the cutting direction. These variables can be regarded as invariants when cutting at a constant speed, so the surface height *R_p_* of the lateral plastic flow profile remains unchanged when cutting at a constant speed. The surface topography model under normal speed cutting conditions and qualitative simulation is also summarized. The expression of the surface topography model of the spindle speed disturbance is expressed as Equation (8).

(8) $$\{\begin{array}{c} y(x)=(1+k)y_{th}(x)\\ k=a+\sum_{n=1}^{\infty }b_{n}\sin(\frac{RVF}{f}2\pi nx+\phi_{n}) \end{array}$$

## 3. Simulation of Workpiece Surface Topography

The factors affecting the surface quality of ultra-precision turning include tool geometry, feed rate, and depth of cut. Considering the formation mechanism of surface topography in the turning process, the main periodic components of the frequency analysis spectrum of the surface profile are composed of the turning feed and spindle rotation errors. If a simulation model of the workpiece surface topography is established, the tool trajectory, cutting parameters, and relative movement between the tool and the workpiece should be comprehensively considered. During ultra-precision machining, process kinematics are defined, machining parameters are set, tool paths and machining process-related conditions are planned, and also the machining results are predicted. With the help of the integrated detection controller, an integrated intelligent system containing monitor, signal process and control is realized. This intelligent system can directly detect the operating state of the spindle system. The result of the coupling effects on the machining process can be achieved, and the surface geometry and workpiece surface topography are indirectly confirmed. 

**Figure 6:**
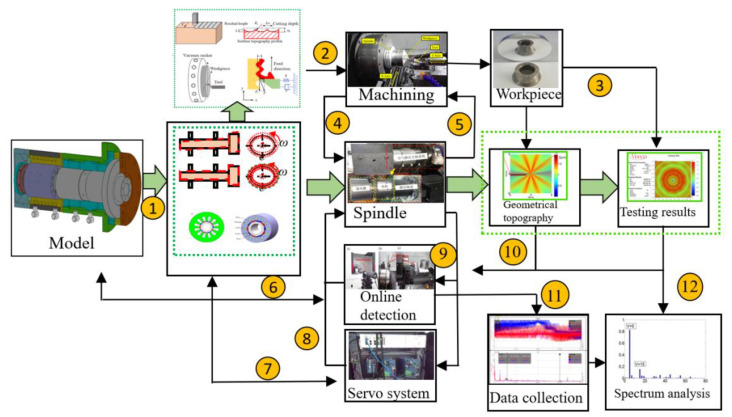
Multi-field coupling analysis and verification process of ultra-precision aerostatic bearing spindle system.

**Figure 7:**
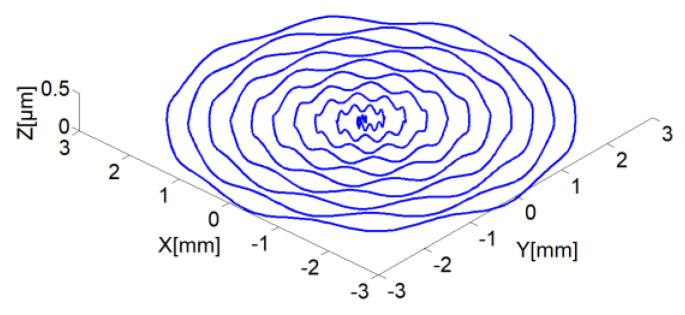
Tool tip trajectory with the tilting motion of the spindle shaft in *Z* axis.

**Figure 8:**
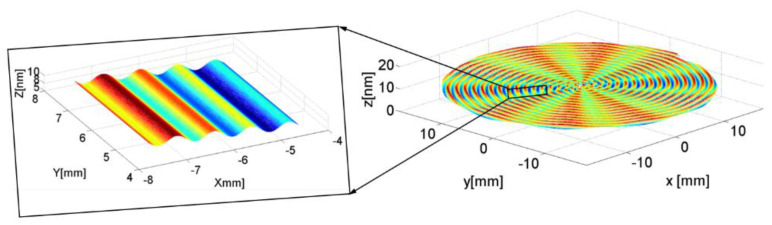
Structure diagram of the workpiece surface topography in cylindrical coordinate system.

The process chart is shown in Figure 6, and the details are as follows: ① Dynamic simulation of ABS, ② cutting process and machining simulation, ③ processing workpiece shape prediction, ④ multi-physics detection, ⑤ autonomous adaptive components, ⑥ self-learning model and simulation of self-calibration detection technology, ⑦ servo control, ⑧ process control, ⑨ integrated control detection, ⑩ workpiece quality data feedback, and automatic adjustment of process settings, ⑪ signal collection library, ⑫ vibration signal multi-frequency spectrum separation calculation, ⑬ vibration frequency spectrum classification, stability. Based on the above analysis, the surface topography of ultra-precision machined workpiece can be predicted and studied. Usually, the surface waviness and texture of the workpiece surface topography are affected by low frequency, mainly due to the rotational vibration of the spindle, feed rate and the vibration of the cutting tool and so on. Meanwhile, based on kinematical-geometrical copying of the cutting tool onto the workpiece surface, the roughness of the workpiece surface are affected by the mechanical properties and thermic preparation, especially the tool position, the working condition of the machine, the cutting force, the tool wear, and the vibrations of the workpiece. Most of them depend on one or more factors predicting the surface roughness. Based on the above analysis, the workpiece surface topography model and qualitative simulation are summarized under the condition of spindle speed perturbation cutting, which is shown in Figure 7. Combined with the actual machining process, the simulation results are shown in Figure 8. The radial partial simulation diagram in the figure is enlarged, and it can be seen that the radial direction produces periodic ripples.

## 4. Turning Experiments

Combining the ultra-precision machining theory, the ultra-precision workpiece surface topography caused by vibrations under the multi-field coupling action of ABS is predicted. By adopting the method of direct contact detection of the spindle performance and mapping workpiece end face, direct inspection is processed. Rotation accuracy data of ABS and workpiece surface geometry, surface quality error data by multi-field coupling, are optimized by the parameters of the main factors affecting the workpiece surface. In order to detect the error of the axial and radial coupling vibrations of ABS, the detection method of ABS with a capacitive sensor mounted on the shaft end, and the turning end face are complementary to detect the coupling axial movement vibrations caused by the spindle shaft rotation. Based on the theoretical research on the sensor detection signal analysis and the simulation model of the workpiece end face process, the comparison and analysis of the processing experiment under different machining parameters, such as different motor rotor eccentricity, cogging motors, skew motors, turning parameters, and different workpiece materials, etc., are carried out. Fourier transform and wavelet analysis techniques are used to conduct multi-scale frequency domain relationship between the signal collected by the signal analyzer and the surface topography, and the corresponding research on the information such as spindle speed, motor rotor eccentricity, and air supply pressure are conducted, so as to obtain more information.

**Figure 9:**
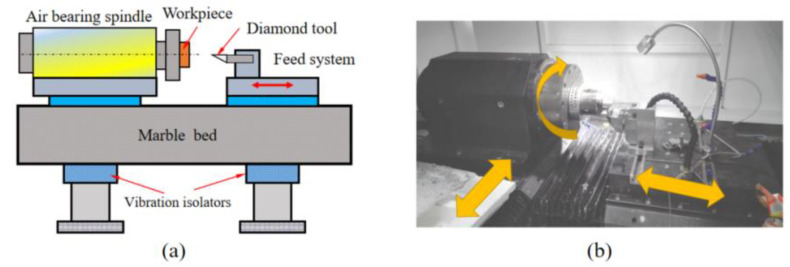
Structure of ultra-precision machine tools: (**a**) 3D model, (**b**) physical machine.

**Figure 10:**
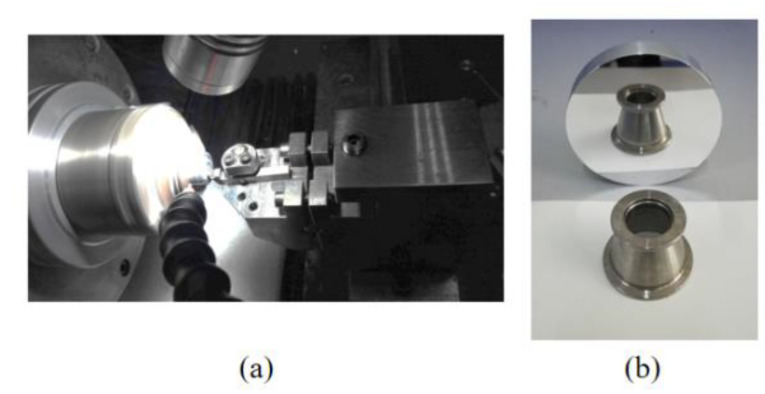
(**a**) Turning experiments of the workpiece surface and (**b**) the machined workpiece surface.

**Figure 11:**
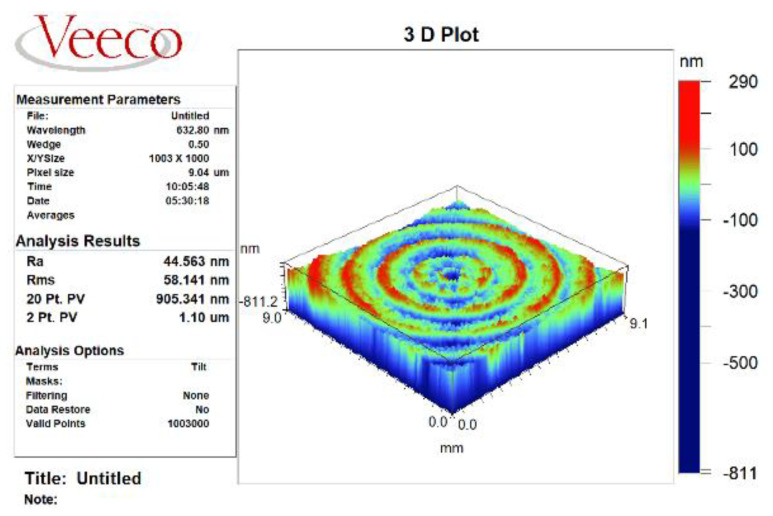
Testing results of the workpiece surface topography.

ABS platform is used for the turning process, the workpiece material is 7075 aluminum alloy, and the diamond tool is used for turning. The experimental platform of ultra-precision turning machine is shown in Figure 9. The machining process experiment is shown in Figure 10a, and the workpiece surface after machining is shown in Figure 10b. The white light interferometer from Zygo (USA) is used to detect the surface quality of the turning process, and the workpiece surface topography is shown in Figure 11. Combined with the test results, it can be seen that the test size is 9.1 mm × 9.1 mm, the surface roughness is *Ra* = 44.56 nm, and the surface profile is *PV* = 1.1 μm. In addition, it can be clearly seen from the figure that the workpiece surface topography presents periodic vibration ripples.

**Figure 12:**
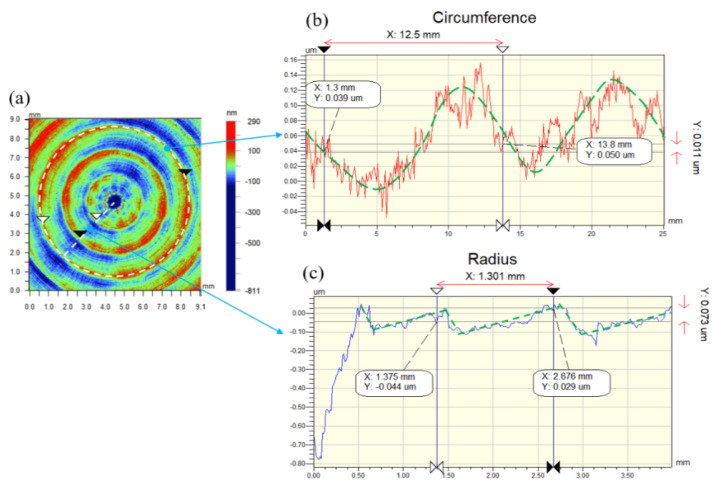
Analysis results of the workpiece surface topography. (**a**) The result of the workpiece surface. (**b**) The quality result when a circle R = 4 mm. (**c**) The quality change of the selected line segment.

The detecting result of the workpiece surface topography is shown in Figure 12. In Figure 12a, a ring with a radius of *R* = 4 mm is selected for the workpiece surface to be tested, and the ring quality is shown in Figure 12b. It can be seen from Figure 12b that the circular ring exhibits periodic vibration in the axial direction, and a frequency of 2 Hz per cycle and a periodic wave arc length of 12.5 mm. At the same time, there are tiny vibration ripples in the circumferential direction. It can be seen from Figure 12b that a line segment is selected from the workpiece center to the edge, the selected line segment produces periodic ripples, and the ripple period distance is 1.3 mm. From the comparison of test results and theoretical analysis, it can be seen that complex vibration ripples are formed in the turning process due to the unstable rotation of ABS. This is caused by the unstable static pressure stiffness and the unbalanced rotation, which cause the turning instability.

**Figure 13:**
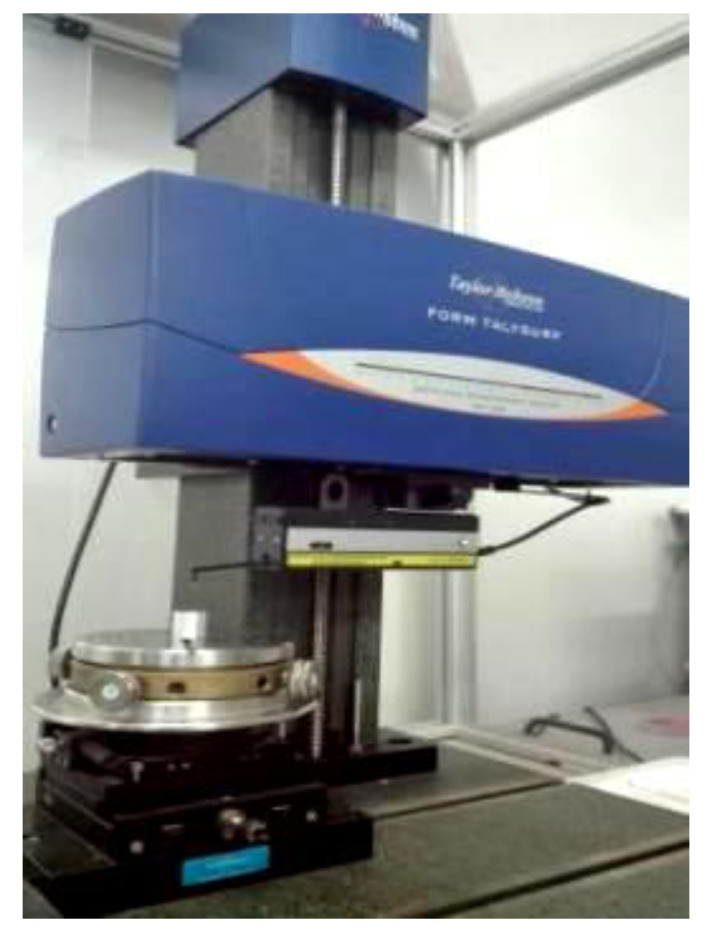
Testing quality of the workpiece surface.

**Figure 14:**
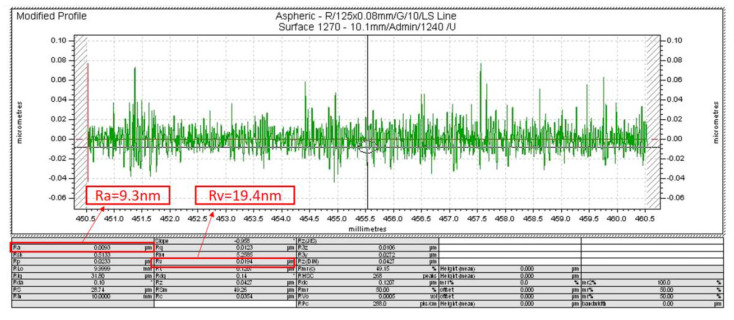
Result of the surface quality.

In addition, the workpiece surface quality is tested with a profiler (Form Talysurf, Taylor Hobson, UK), and the testing result process is shown in Figure 13. The surface quality is shown in Figure 14. It can be seen from the figure that the surface roughness *Ra* = 9.3 nm, the maximum profile valley depth *Rv* = 19.3 nm, and the homemade UPAMTM has good performance.

## 5. Conclusions

Ultra-precision turning process is a complicated process, and the analysis of the workpiece surface-forming mechanism has important research significance. In this paper, the mechanism of multi-field coupling vibration of ABS is revealed, and the influence law between the tool vibration and the turning shape ripples, proposes the separation method of the multi-field coupling vibration frequency spectrum, and obtains the mapping relationship of each coupling factor. The experimental results show that the model can effectively express the surface topography under the condition of the spindle speed disturbance when the initial cutting state is stable, and the model parameters describing the surface topography and the spindle speed disturbance parameters have a linear correlation. 

The influence law of the coupling vibration error of ABS under the multi-field coupling action on the cutting surface topography is revealed. It reveals the law of the influence of coupled vibration on the turning shape and the law of the influence of tool vibration on the turning shape. The law of the forming surface texture is verified through machined workpiece surface and tests by a homemade ultraprecision system. The end face of the workpiece forms periodically fluctuating wave and ripple patterns, and the comparisons between theoretical analysis and experimental detection of the surface topography is verified.
